# A Diagnostic Scoring Model for Leptospirosis in Resource Limited Settings

**DOI:** 10.1371/journal.pntd.0004513

**Published:** 2016-06-22

**Authors:** Senaka Rajapakse, Praveen Weeratunga, Roshan Niloofa, Narmada Fernando, Nipun Lakshitha de Silva, Chaturaka Rodrigo, Sachith Maduranga, Nuwanthi Nandasiri, Sunil Premawansa, Lilani Karunanayake, H. Janaka de Silva, Shiroma Handunnetti

**Affiliations:** 1 Department of Clinical Medicine, Faculty of Medicine, University of Colombo, Sri Lanka; 2 University Medical Unit, National Hospital of Sri Lanka, Colombo; 3 Institute of Biochemistry, Molecular Biology and Biotechnology, University of Colombo, Sri Lanka; 4 Sir John Kotelawala Defence University, Sri Lanka; 5 Ministry of Health, Sri Lanka; 6 Department of Zoology, Faculty of Science, University of Colombo, Sri Lanka; 7 Medical Research Institute, Colombo, Sri Lanka; 8 Department of Medicine, Faculty of Medicine, University of Kelaniya, Ragama, Sri Lanka; Institute of Collective Health, Federal University of Bahia, BRAZIL

## Abstract

**Background:**

Leptospirosis is a zoonotic infection with significant morbidity and mortality. The clinical presentation of leptospirosis is known to mimic the clinical profile of other prevalent tropical fevers. Laboratory confirmation of leptospirosis is based on the reference standard microscopic agglutination test (MAT), direct demonstration of the organism, and isolation by culture and DNA detection by polymerase chain reaction (PCR) amplification. However these methods of confirmation are not widely available in resource limited settings where the infection is prevalent, and reliance is placed on clinical features for provisional diagnosis. In this prospective study, we attempted to develop a model for diagnosis of leptospirosis, based on clinical features and standard laboratory test results.

**Methods:**

The diagnostic score was developed based on data from a prospective multicentre study in two hospitals in the Western Province of Sri Lanka. All patients presenting to these hospitals with a suspected diagnosis of leptospirosis, based on the WHO surveillance criteria, were recruited. Confirmed disease was defined as positive genus specific MAT (*Leptospira biflexa*). A derivation cohort and a validation cohort were randomly selected from available data. Clinical and laboratory manifestations associated with confirmed leptospirosis in the derivation cohort were selected for construction of a multivariate regression model with correlation matrices, and adjusted odds ratios were extracted for significant variables. The odds ratios thus derived were subsequently utilized in the criteria model, and sensitivity and specificity examined with ROC curves.

**Results:**

A total of 592 patients were included in the final analysis with 450 (180 confirmed leptospirosis) in the derivation cohort and 142 (52 confirmed leptospirosis) in the validation cohort. The variables in the final model were: history of exposure to a possible source of leptospirosis (adjusted OR = 2.827; 95% CI = 1.517–5.435; p = 0.001) serum creatinine > 150 micromol/l (adjusted OR = 2.735; 95% CI = 1.374–4.901; p = 0.001), neutrophil differential percentage > 80.0% of total white blood cell count (adjusted OR 2.163; 95% CI = 1.309–3.847; p = 0.032), serum bilirubin > 30 micromol/l (adjusted OR = 1.717; 95% CI 0.938–3.456; p = 0.049) and platelet count < 85,000/mm^3^ (adjusted OR = 2.350; 95% CI = 1.481–4.513; p = 0.006). Hosmer-Lemeshow test for goodness of fit was 0.931. The Nagelkerke R2 was 0.622. The area under the curve (AUC) was noted as 0.762. A score value of 14 reflected a sensitivity of 0.803, specificity of 0.602, a PPV of 0.54, NPV of 0.84, a positive LR of 2.01 and a negative LR of 0.32.

**Conclusions:**

The above diagnostic model for diagnosis of leptospirosis is suggested for use in clinical settings. It should be further validated in clinical practice.

## Author Summary

Leptospirosis is a bacterial disease which is common in tropical countries, and spreads via contaminated rat urine. It has a potential for mortality and can cause failure of multiple body systems. The features of the disease mimics several other tropical diseases prevalent in the region. The modalities for laboratory confirmation of the disease are not readily available in resource limited settings. This study utilizes prospective data and proposes diagnostic criteria using simple clinical presentations and easily accessible laboratory data.

## Introduction

Leptospirosis is a zoonotic infection caused by spirochaetes of the genus *Leptospira*, with humans being affected as incidental hosts. Infection occurs when water or soil contaminated with urine of infected animals (commonly rodents) comes into contact with abraded human skin or mucous membrane. Clinically, leptospirosis infection has a range of manifestations, from a mild febrile illness to a severe and potentially fatal disease with acute kidney injury, liver dysfunction, pulmonary haemorrhage and acute respiratory distress syndrome, bleeding, and cardiac involvement. The burden of leptospirosis is high; the WHO Leptospirosis Burden Epidemiology Reference Group (LERG) estimates 873,000 annual cases and 48,000 deaths due to leptospirosis[1]. Leptospirosis is endemic in Sri Lanka. Data from the Epidemiology Unit of the Ministry of Health, Sri Lanka, suggests that leptospirosis has emerged as a key infection over the last 20 years. From 167 cases in 1991, the numbers have increased to 4545 cases in 2010[2].

The clinical manifestations of leptospirosis mimic those of several other tropical diseases including dengue, Hanta-virus infection, rickettsial infection, as well as bacterial sepsis. In many areas where leptospirosis is common, there is also a high incidence of viral infections, including haemorrhagic fevers and infections which result in organ dysfunction. There is often confusion in differentiating leptospirosis from other infections, particularly dengue, during high incidence periods[3, 4]. Differentiating leptospirosis from these other diseases is often a challenge to the clinician. The laboratory confirmation of leptospirosis is often based on the microscopic agglutination test (MAT), isolation of the organism, or demonstration of leptospiral DNA by means of PCR. While these tests are useful for epidemiological purposes, they are not available freely, or available in a timely manner, to clinicians treating cases of acute febrile illness. For example, in Sri Lanka, where the incidence of leptospirosis, dengue, and rickettsial infections are high, reliance on specific diagnostics is impractical due to lack of availability of tests, and also because of possible cross-reacting antibodies. MAT is performed only in one reference laboratory in the country. PCR is not widely available, and rapid immunodiagnostics are expensive and not widely validated. Clinicians often depend on clinical features to diagnose and treat these conditions, based on available guidelines and clinical experience.

In this study, we prospectively evaluated patients presenting to hospital with a suspected diagnosis of leptospirosis, in order to determine the clinical and investigation characteristics which may help differentiate leptospirosis from other infections presenting with a similar clinical picture, and attempted to develop a model for diagnosis of leptospirosis.

## Methods

### Study design and population

Data were collected as a part of prospective study carried out in two hospitals in the Western province of Sri Lanka, which is one of the high prevalence areas for leptospirosis in Sri Lanka. An analysis of hospital based sentinel surveillance data of leptospirosis over 4 years in Sri Lanka has confirmed that, of nearly 4000 suspected cases, 47% were from this province[5]. The selected hospitals were the National Hospital of Sri Lanka (NHSL) and the Homagama Base Hospital. The NHSL is the apex tertiary care teaching hospital in Sri Lanka where severely ill patients including those with complicated leptospirosis are transferred for further treatment. The Homagama base hospital is located in an area highly endemic for leptospirosis. Data collection was performed over a period of 24 months starting from June 2012.

All patients aged over 12 years with a suspected diagnosis of leptospirosis, based on the WHO surveillance criteria[6], were enrolled. The clinical criteria for enrolment were: acute febrile illness with headache, myalgia and prostration, with any of the following—conjunctival suffusion, jaundice, oliguria/haematuria, cardiac arrhythmia or failure, cough, haemoptysis & breathlessness, bleeding, features of meningeal irritation, skin rash, history of exposure to potentially contaminated water or soil. Patients with a definite alternative diagnosis available at the time of admission were excluded. Data collection was by research assistants independent of the primary treating teams. Clinical features and investigation findings were recorded on a daily basis until the point of discharge or death. A total of 600 patients were included, with random allocation of 450 patients into a derivation cohort and 150 patients into a validation cohort.

### Laboratory confirmation of disease and classification as leptospirosis or non-leptospirosis fever

Microscopic Agglutination Test (MAT) was performed at the Medical Research Institute, Colombo, for laboratory confirmation of the leptospirosis. A positive result in MAT was considered under three circumstances; i.e., a) MAT titer of 400 or greater in single or paired samples, b) a four-fold increase in MAT titer between acute and convalescent serum samples, or c) seroconversion to a MAT titer greater than 200 between paired sampling[6]. Patients were classified as leptospirosis or non-leptospirosis fever (NLF) based on laboratory confirmation, retrospectively. The research assistants collecting data were blinded to the results of the confirmatory tests. The MAT used was based on the genus specific *Leptospira biflexa* serovar Patoc strain Patoc-1.

### Development of the diagnostic scoring model

A derivation cohort was selected from the available data using a random sample of 450 out of the total 592 patients. Univariate analysis was performed on the derivation cohort with formulation of clinical, demographic, epidemiological and laboratory criteria associated with MAT positivity.

All numerical data for clinical and laboratory values were plotted and tested for normality with histograms and superimposed curves. The mean values were selected for dichotomization and cut-off points for the data conforming to a normal distribution. The median values were selected in the case of non-normal data distributions. These cut-off points were subsequently refined and rounded to the closest integer with clinical application. All clinical and laboratory data extracted for analysis and subsequent model construction were the first available parameters on admission. Day three of illness was the mean day of admission.

A backward multivariate logistic regression model was applied to derive the variables for the final diagnostic predictive model. All variables with p < 0.2 were utilized in the multivariate model. Significant independent predictors from this model were identified and their coefficients were examined. The goodness of fit of the model was analysed using the Hosmer-Lemeshow statistic. Furthermore, the Nagelkerke R2 was calculated.

### Validation of the scoring model

In order to obtain a practical scoring model, all coefficients were divided by the smallest coefficient and rounded to the closest integer (fractions of 0.5 and above were rounded to the higher number, while those below 0.5 were rounded down). The scoring model was validated in the remaining patients (i.e., those not selected randomly for the derivation cohort). ROC curves were generated to examine the sensitivity and specificity of the model with MAT positivity and confirmation of the disease denoted as the gold standard.

### Ethical statement

Ethics clearance was obtained from the Ethics Review Committee of the Faculty of Medicine, University of Colombo (EC-12-056) and the Ethics Review Committee of the NHSL. Informed written consent was obtained from all the participants prior to recruitment to the study.

## Results

**Fig 1:**
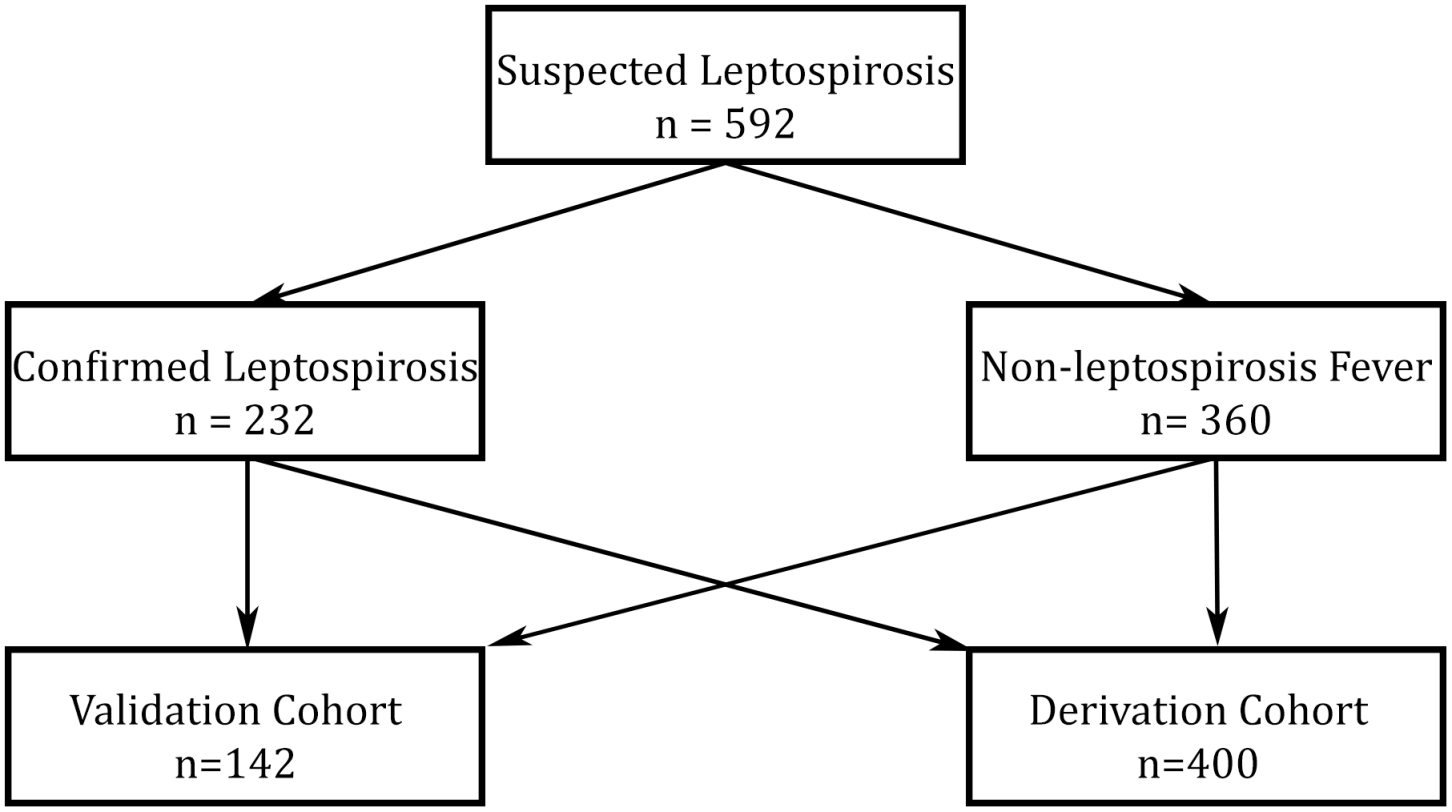
Schematic flow diagram of study subject categorization.

A total of 592 patients were included in the final analysis with 450 in the derivation cohort and 142 in the validation cohort. In the derivation cohort, there were 180 with confirmed leptospirosis, and 270 with non-leptospirosis fever. In the validation cohort there were 52 with confirmed leptospirosis and 90 with non-leptospirosis fever (Fig 1). Socio-demographic, clinical and laboratory characteristics of the validation cohort and derivation cohort were similar.

Overall, there were 232 patients with confirmed leptospirosis, from a total of 592 admitted with suspected leptospirosis (39.2%). The mean age for leptospirosis patients was 42.36 years, and that for NLF was 42.97 years. The mean ages of patients with leptospirosis in the derivation cohort and the validation cohort were 41.95 years and 42.25 years respectively.

The presenting characteristics, clinical and laboratory profiles of patients was uniform between the included healthcare institutions (Table 1).

**Table 1:** Distribution of patients within hospitals.

Disease classification	National Hospital of Sri Lanka	District General Hospital, Homagama	Total
Leptospirosis	192 (82.75%)	40 (17.25%)	232
Dengue fever	150 (85.71%)	25 (14.29%)	175
Respiratory tract infection	76 (74.50%)	20 (26.50%)	102
Urinary tract infection	44 (72.13%)	17 (27.87%)	61
Gastrointestinal infection	14 (87.5%)	2 (12.5%)	16
CNS infection	1 (100%)	0 (0%)	1
Other	1 (20%)	4 (80%)	5

The male: female ratio was 86.6:13.4 for leptospirosis and 87.5:12.5 for NLF. The presenting features and laboratory criteria of patients with leptospirosis and NLF are compared in Table 2. The majority of patients included were from the National Hospital of Sri Lanka, with 192 patients with confirmed leptospirosis and 286 patients with NLF.

**Table 2:** Management and outcomes in patients in leptospirosis and non- leptospirosis fever.

	Leptospirosis	Non leptospirosis fever	P value
**Use of antibiotics**	232 (100%)	180 (50%)	0.047*
**Requirement for renal replacement therapy**	135 (58.19%)	6 (1.67%)	0.001*
**Requirement for mechanical ventilation**	4 (1.72%)	3 (0.833)	0.452
**Discharge**	220 (94.82%)	350 (97.22%)	0.584
**Death**	7 (3.01%)	7 (1.94%)	0.235
**Transfer**	5 (2.15%)	3 (0.83%)	0.665

*Significant at a level of p < 0.05

The management data and outcomes are presented in Table 2. A higher proportion of patients with leptospirosis required antibiotics (p = 0.047) and renal replacement therapy (p = 0.001) when compared to patients with NLF. The mortality rates among patients with leptospirosis and NLF were 3.01% and 1.90% respectively. Multi-organ failure was the most common cause of death in patients with leptospirosis.

MAT was noted to positive in 232 patients, of which 85 (36.63%) were patients with sero-conversion to a MAT titre greater than 200 between paired sampling. A four-fold increase in MAT titre between acute and convalescent serum samples was noted in 77 patients (33.19%). Seventy (30.17%) patients had a MAT titre of 400 or greater in single or paired samples. The geometric mean titre was 800.

### Derivation cohort

The results of the univariate analysis of associations of MAT positive, confirmed cases of leptospirosis are depicted in Table 3. The following were positively associated with confirmed leptospirosis at a significance level of p < 0.05: history of exposure, myalgia, conjunctival suffusion, oliguria, acute kidney injury, urea > 18 mmol/l (normal range [NR] 2.9–8.2), serum creatinine > 150 micromol/l (NR 60–120), bilirubin concentration > 30 micromol/l (NR 5–21), serum sodium concentration < 130 mEq/l (NR 135–148), total white blood cell (WBC) count > 11500/mm^3^ (4000–7000) with neutrophil percentage > 80%, haemoglobin concentration < 10.5g/dL (NR 11–12) and packed cell volume < 30% and platelet count < 85000/mm^3^ (NR 150,000–450,000).

**Table 3:** Comparison of clinical and laboratory features in leptospirosis and NLF patients in the derivation cohort (univariate analysis).

Characteristic	Derivation cohort (n = 450)		P value
	Leptospirosis (n = 180)	Non-leptospirosis fever (n = 270)	
Fever	171 (95%)	253 (93.7%)	0.846
Rigors	133 (73.9%)	188 (69.6%)	0.907
Exposure	91 (74.6%)	115 (53.0%)	0.000*
Headache	147 (81.7%)	209 (77.4%)	0.816
Myalgia	159 (88.3%)	216 (80.0%)	0.025*
Conjunctival Suffusion	99 (55%)	117 (43.3%)	0.048*
Jaundice	49 (27.2%)	48 (17.8%)	0.199
Photophobia	16 (8.9%)	18 (6.7%)	0.220
Neck Stiffness	24 (13.3%)	37 (13.7%)	0.852
Muscle Tenderness	122 (67.8%)	167 (61.9%)	0.382
Chest Pain	38 (21.1%)	49 (18.1%)	0.841
Oliguria	56 (31.1%)	50 (18.5%)	0.007*
Smoking	53 (29.4%)	97 (35.9%)	0.270
Alcohol	57 (31.7%)	88 (32.6%)	0.840
Acute Kidney Injury	118 (65.6%)	76 (28.1%)	0.000*
Myocarditis	4 (2.2%)	3 (1.1%)	0.678
Acute liver injury	2 (1.6%)	2 (0.9%)	0.518
ARDS	3 (1.7%)	3 (1.1%)	0.105
Pulmonary Haemorrhage	1 (0.6%)	0	0.253
Shock	9 (5.0%)	9 (3.3%)	0.571
Multi Organ Dysfunction	1 (0.6%)	3 (1.1%)	0.578
Urea Highest > 18	43 (23.8%)	19 (7.0%)	0.000*
Creatinine Highest > 150	122 (67.8%)	81 (30%)	0.000*
Na^+^ Highest > 140	95 (52.7%)	108 (40.0%)	0.045
Na^+^ Lowest < 130	85 (47.2%)	78 (28.9%)	0.045
K^+^ Highest > 4.2	96 (53.3%)	113 (41.9%)	0.089
K^+^ Lowest < 3.5	66 (36.7%)	60 (22.2%)	0.228
Bilirubin Highest > 30	77 (41.1%)	51 (18.9%)	0.000
ALT Highest > 60	72 (40.6%)	74 (27.4%)	0.092
AST Highest > 70	68 (36.7%)	77 (28.5%)	0.977
WBC Highest > 11500	104 (57.8%)	113 (41.9%)	0.018*
WBC Lowest <7000	61 (33.9%)	122 (45.2%)	0.010*
Neutrophils Highest > 80%	110 (61.1%)	107 (39.6%)	0.000*
Neutrophils Lowest < 70.0%	65 (36.1%)	119 (44.1%)	0.452
Haemoglobin Highest > 12.0	66 (36.7%)	148 (54.8%)	0.012*
Haemoglobin Lowest < 10.5	90 (50.0%)	95 (35.2%)	0.038*
PCV Highest > 36	65 (36.1%)	146 (54.1%)	0.014*
PCV Lowest < 30	98 (54.4%)	91 (33.1%)	0.000*
Platelets Lowest < 85000	99 (55.0%)	87 (32.2%)	0.012*

*Significant at a level of p < 0.05

Table 3. Comparison of clinical and laboratory features in leptospirosis and NLF patients in the derivation cohort (univariate analysis)

The selection criteria of independent variables for model construction were described under methodology. The presence of conjunctival suffusion, jaundice, exposure history, muscle tenderness, total WBC count > 11,500mm^3^, neutrophil percentage > 80.0%, serum creatinine >150micromol/l, bilirubin > 30 micromol/l, hemoglobin < 10.5g/dL, serum sodium < 130 mEq/L, ALT > 70 IU/L (NR– 10–35), microscopic hematuria, and serum potassium > 5.0 (NR– 3.5–5.3) were entered into the initial model. Correlation matrices were used to adjust for co-dependence between the independent variables. Contact history was adjusted for sex and age, serum creatinine was adjusted for age, and haemoglobin for sex.

The variables in the final step model were: history of exposure to possible source of leptospirosis (adjusted OR = 2.827; 95% CI = 1.517–5.435; p = 0.001), serum creatinine > 150 micromol/l (adjusted OR = 2.735; 95% CI = 1.374–4.901; p = 0.001), neutrophil differential percentage > 80.0% of total WBC count (adjusted OR 2.163; 95% CI = 1.309–3.847; p = 0.032), serum bilirubin > 30 micromol/l (adjusted OR = 1.717; 95% CI 0.938–3.456; p = 0.049) and platelet count < 85,000/mm^3^ (adjusted OR = 2.350; 95% CI = 1.481–4.513; p = 0.006). Hosmer-Lemeshow test for goodness of fit was 0.931. The Nagelkerke R2 was 0.622

The diagnostic score derived from the above is shown in Table 4. The beta coefficients were divided by the smallest coefficient and then multiplied by a factor of 4 to create a more robust and practical scoring system.

**Table 4:** Proposed score for diagnosis of leptospirosis, based on multivariate analysis.

Characteristic	Beta coefficient	Calculated score	Score (rounded to nearest integer)
Bilirubin > 30	0.562	4.0	4
Neutrophil > 80	0.724	5.15	5
Exposure	1.057	7.52	8
Serum creatinine >150	1.009	7.18	7
Platelet < 85,000	0.854	6.07	6
Maximum possible score			**30**

### Validation of the scoring system

**Fig 2:**
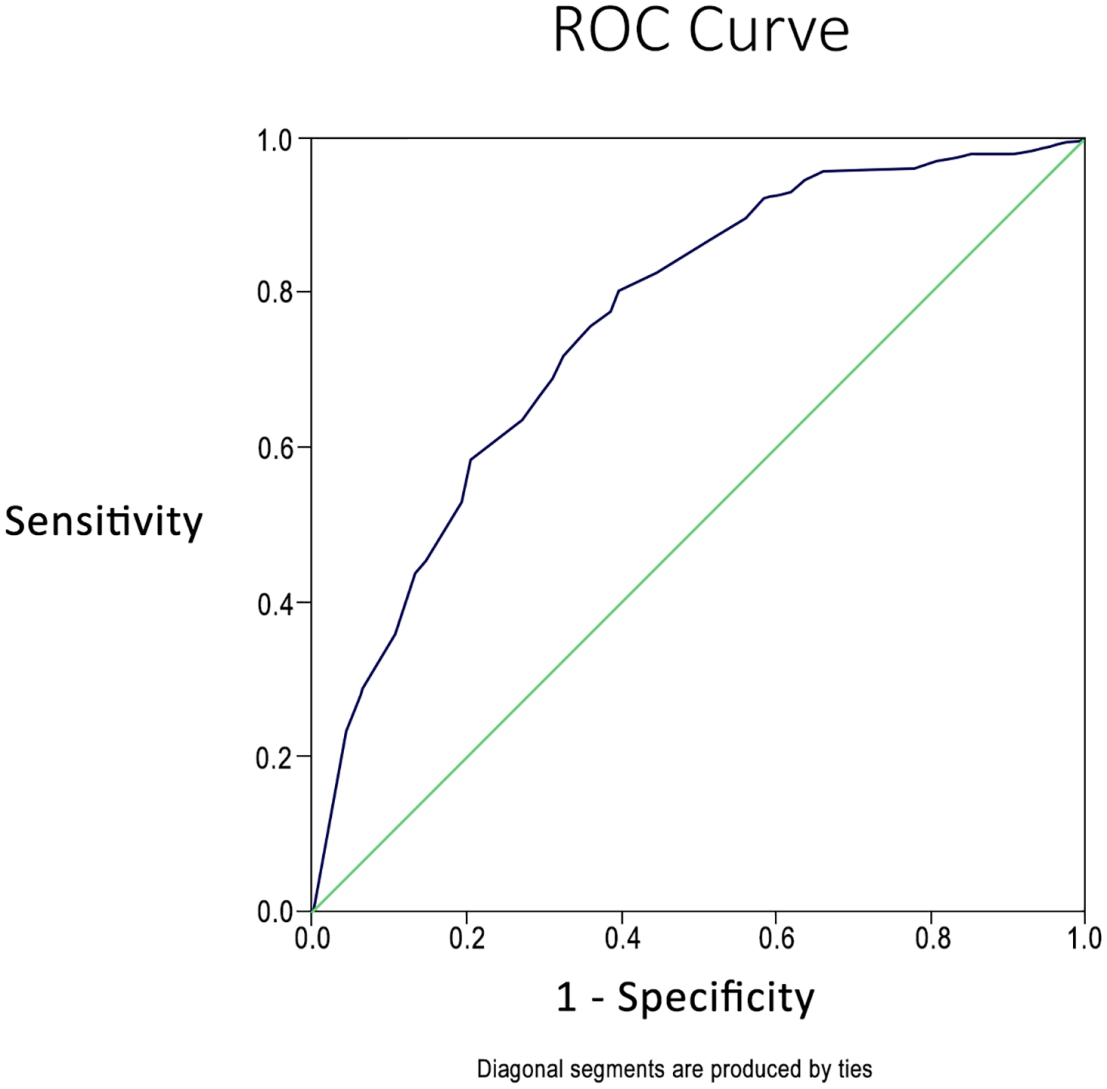
Co-ordinates of the ROC curve for outcome—positive MAT.

Receiver operating characteristic (ROC) curves were generated separately for serum creatinine, neutrophil differential percentage, serum bilirubin, and platelet count. Furthermore, we generated an ROC curve utilizing the scoring system applied to the validation cohort to differentiate leptospirosis from NLF (Fig 2). The sensitivity, specificity, positive predictive value (PPV), negative predictive value (NPV) and likelihood ratios (LR) are presented. The area under the curve (AUC) was noted as 0.762. A score value of 14 reflected a sensitivity of 0.803, specificity of 0.602, a PPV of 0.54, NPV of 0.84, a positive LR of 2.01 and a negative LR of 0.32. The diagnostic model performance parameters for various cut-off points are presented in Table 5. All coordinates in the ROC curve with relevant sensitivity and specificity are presented in S1 Table.

**Table 5:** Diagnostic model performance (sensitivity, specificity, positive and negative predictive value and likelihood ratios).

Score	Sensitivity	Specificity	PPV	NPV	Positive LR	Negative LR
5	0.979	0.096	0.38	0.88	1.08	0.21
10	0.944	0.367	0.46	0.91	1.49	0.15
12	0.873	0.476	0.49	0.86	1.66	0.26
14	0.803	0.602	0.54	0.84	2.01	0.32
15	0.775	0.614	0.54	0.82	2.0	0.37
20	0.585	0.795	0.62	0.76	2.85	0.52
25	0.289	0.934	0.71	0.69	4.3	0.76

The dataset is presented as S1 Dataset.

## Discussion

The incidence of confirmed leptospirosis is high among patients admitted to hospital with a suggestive clinical picture. However nearly 52% of patients with suspected leptospirosis were negative on confirmatory testing. Nonetheless, since confirmatory test results are often delayed, the majority of them were treated with appropriate antibiotics to cover leptospirosis; in our study, this was nearly 100% in those with a contact history of leptospirosis. Our results indicates that a diagnostic model with inclusion of serum creatinine, neutrophil percentage, elevated serum bilirubin and platelet count has reasonably good sensitivity and specificity for the diagnosis of leptospirosis. Notably, apart from exposure, none of the clinical features looked for could reliably distinguish leptospirosis from NLF. This reinforces the need for accurate and readily available tests to confirm the diagnosis.

History of exposure was noted to have the strongest positive association. Thus, exploring the different sources of exposure is one of the most useful components of the clinical history. An open ended question asking whether there is exposure to muddy water maybe inadequate, and we suggest developing a list of potential exposures that should be asked for at the time of admission.

Specific organ involvement, i.e., kidney and liver, appear to differentiate leptospirosis from NLF. Haematological parameters are of particular use, since a full blood count is often the first investigation to become available. A low platelet count, while often the hallmark of dengue, also appears to be a feature of leptospirosis. The absolute neutrophil percentage appears to be a more useful indicator of leptospirosis rather than the total leucocyte count.

Faine’s criteria [7] for the diagnosis of leptospirosis have been suggested for the diagnosis of leptospirosis, with various subsequent modifications[8]. Faine’s criteria essentially use clinical, epidemiological and microbiological features to score the likelihood of leptospirosis. These criteria, with modifications, have been evaluated in various studies, giving varying degrees of specificity and sensitivity [8, 9]. The use of clinical criteria alone was found to have high negative predictive value but relatively low positive predictive value; however studies have been small [9, 10]. The numbers included in our study were greater, and a large panel of clinical and laboratory characteristics were evaluated in our diagnostic model.

The patients utilized in derivation of this diagnostic model were hospitalized patients and the data may not be universally applicable to patients with milder disease and outpatients presenting with acute febrile illness.

Our study has certain limitations. First, the final diagnosis of patients in the NLF category was not available in all cases. This would have created better characterization between other leptospirosis mimics. Secondly, with the high awareness of dengue and the wide availability of Dengue NS1 antigen testing, many patients admitted with a similar clinical picture present to hospital with positive results of dengue NS1 antigen, and would thus have been excluded from the study. Thirdly, the MAT methodology available up to 2015 in Sri Lanka was limited to the Patoc serovar analysis. While cross reactivity between pathogenic serovars and the saprophytic serovars occurs, it is possible that there were false negatives on MAT testing. Further analysis of the study population with a broader panel of pathogenic serovars has been initiated. Finally, the use of MAT as a gold standard has been questioned in prior studies [11] where estimated sensitivity of MAT and MAT + culture is noted to be less than 50%. Nonetheless, this scoring system applies to the optimum currently available laboratory standards in resource limited settings such as Sri Lanka.

We suggest the use of the above diagnostic model for the diagnosis of leptospirosis in clinical settings. This model should be further validated in clinical practice, and with a broader panel of serovars.

## Supporting Information

S1 Checklist STROBE Checklist. (DOCX)

S1 Table Coordinates of the ROC curve. (XLSX)

S1 Dataset Dataset utilized for analysis. (SAV)
