# Supplementary material for: A Diagnostic Scoring Model for Leptospirosis in Resource Limited Settings
Source: PLoS Negl Trop Dis. 2016 Jun 22;10(6):e0004513. doi: 10.1371/journal.pntd.0004513 (PMC4917116; doi:10.1371/journal.pntd.0004513)
Supplement: S1 Checklist — (DOCX) [file pntd.0004513.s001.docx]

STROBE Statement—checklist of items that should be included in reports of observational studies

|  | Item No | Page and section number in the manuscript |
| --- | --- | --- |
| **Title and abstract** | 1 | Page 1, 2 and 3 |
|  |  |  |
| Introduction | | |
| Background/rationale | 2 | Page 4 |
| Objectives | 3 | Page 5 |
| Methods | | |
| Study design | 4 | Page 5 paragraph 3 |
| Setting | 5 | Page 5 paragraph 3 |
| Participants | 6 | Page 5 paragraph 4 |
|  |  |  |
| Variables | 7 | Page 6 |
| Data sources/ measurement | 8* | Page 5 and page 6 |
| Bias | 9 | None |
| Study size | 10 | Page 5 |
| Quantitative variables | 11 | Page 6 |
| Statistical methods | 12 | Page 6 paragraph 4 and page 7 paragraph 1 |
|  |  |  |
|  |  |  |
|  |  |  |
|  |  |  |

Continued on next page

| Results | | |
| --- | --- | --- |
| Participants | 13* | Page 7 paragraph 4 |
|  |  | Figures |
|  |  |  |
| Descriptive data | 14* | Page 7 paragraph 4-7 |
|  |  |  |
|  |  |  |
| Outcome data | 15* | Page 8 paragraph 1 |
|  |  |  |
|  |  |  |
| Main results | 16 | Pages 8 to 9 |
|  |  |  |
|  |  |  |
| Other analyses | 17 | Page 9 |
| Discussion | | |
| Key results | 18 | Page 9 |
| Limitations | 19 | Page 10 |
| Interpretation | 20 | Page 10-11 |
| Generalisability | 21 | Page 10-11 |
| Other information | | |
| Funding | 22 | In submission file |
